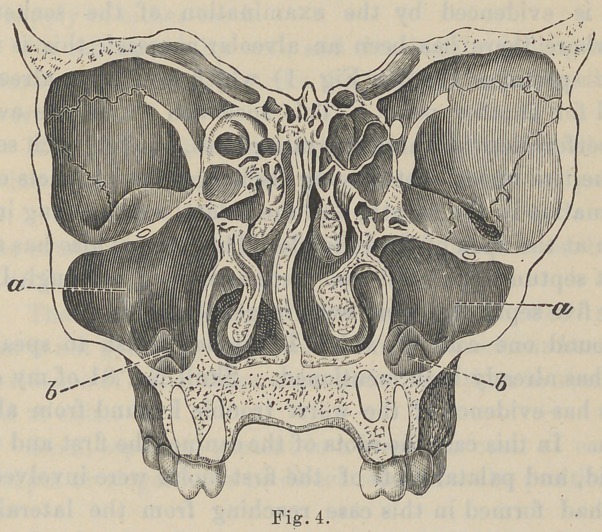# Some Suggestions as to the Relation of the Teeth to Empyema of the Maxillary Sinus

**Published:** 1894-03

**Authors:** M. H. Fletcher

**Affiliations:** Cincinnati, Ohio


### THE DENTAL REGISTER.

VOL. XLVIII.] MARCH, 1894. [No. 3.

### COMMUNICATIONS.

### Some Suggestions as to the Relation of the Teeth to Empyema of the Maxillary Sinus.

BY M. H. FLETCHER, M.D., CINCINNATI, OHIO.

Read in the Sect'on on D ntal and Oral Surgery, at the Forty-fourth Annual Meeting of the American Medical Association.

In this paper the anatomy, pathology and treatment, as usually given on disease of the antrum of Highmore, will be largely omitted, since the disease of this cavity is a subject much written upon these points must be familiar to all who have given the subject any considerable attention. It is desired, however, to deal principally with the etiology, believing that if the cause of the disease is correctly diagnosed, the treatment is comparatively easy.

Authors and practitioners seem to be divided into two classes, viz., those who believe that diseases of the maxillary sinus are most frequently caused by dental lesions, and those who believe that they are more largely due to intra-nasal disorders. The latter view is held by Zuckerkandl, Schiffert, Chatallier, Krause and Bosworth.

Those who look more largely to the teeth as a cause of trouble are Lewis, Baratoux, Noquet, Boucheron, Garel, Gele, Beverly Robinson, Lennox Browne, Garretson and Tiffany. In "American System of Dentistry" (page 562), Tiffany says in regard to disease of the antrum: "It is not met with as an idiopathic affection; it occurs as the result of injury and as an extension from a diseased tooth. The first and second molars are the most likely to act as the inciting causes, as their fangs *project into the floor of the antrum.*

In treating of the diseases of the antrum, Lennox Browne says: "*The cause is almost invariably an alveolar abscess*, which has extended into the antrum through a natural connection with that cavity—as in the case of the first molar tooth or breaking down of the slight bony partition in some of the other upper teeth."

Bryant in his last edition says: "Suppuration of this cavity is often due, doubtless, to the extension of inflammation from the teeth."

These quotations are a fair sample of the words of most of those men who claim that antral troubles come largely from the teeth, and it would seem that they copy largely one from another. This I deem to be a defect in many of our text-books; many men copy from previous writers or write *for* experience instead of *from* experience, thus leaving us without additional knowledge.

Those writers of contrary opinion cite many pathologic conditions of the intra-nasal tissues as the more frequent cause. Among these causes are new growths, catarrhal inflammation, acute and chronic, and stenosis of the osteum maxillare.

It is not proposed to go largely into the etiology of the intra-nasal troubles, but give attention more especially to the claims of those who blame the teeth for these disorders. The writer's views coincide with those of Zuckerkandl, Schiffert and others who believe these antral troubles are more largely caused by intra-nasal disorders, and I wish herewith to present evidence for such an opinion.

This opinion has been arrived at after some years of experience, in addition to the examination of *one hundred skulls.* These skulls were examined with special reference to the relation of diseases of the teeth to the antrum. It is known that the anatomy of the antra of the superior maxillary bones and their nervous supply is such that there can be no disease by reflex action from one to the other as occurs, for instance, in sympathetic ophthalmia, consequently these cavities are dealt with as separate organs. The statistics then would show two hundred antra, instead of one hundred. These skulls were examined for five points, viz., 1, for abscessed teeth; 2, for septa; 3, for conical protrusion of the roots of the teeth into the antrum; 4, for perforation by the roots of the teeth without protrusion; 5, for perforation of the antrum from ulcerated teeth.

Fig. 1.

1. As to abscessed teeth and the connection of such abscesses with the antrum. Only such teeth are mentioned as might most easily produce antral trouble, viz., the three upper molars, the bicuspids and canines being too far forward to be counted in these statistics. I might say, however, that in a number of these skulls evidence of ulceration was found in the bicuspids and canines, with no apparent connection whatever with the antra, save in one case. As to the molar teeth, ulceration was found in more than 25 per cent. of the cases; there being in these two hundred examinations, fifty-seven ulcerated teeth, and out of these *fifty-seven possible cases* of perforation by inflammation and its results, we found such to be the case *only four times;* all other cases having perforated the alveolar border and discharged the pus into the mouth, two of them discharging both in the mouth

Fig. 2.

and in the antrum, as is evidenced by the specimen No. 1. (See Fig. 1-b)*

This does not show a very large proportion of cases where antral trouble has come from the teeth, being less than 8 per cent. in fifty-seven possible cases.

In addition to these figures, I wish to offer as negative evidenc, statistics from my own records in regard to the relation of these diseases of the teeth to the antrum, as they have come under the writer's observation. I have in the past 10 years treated 916 cases of pulpless teeth. Two hundred and twenty-four of these being superior molars, which, according to the authors named above, could and probably would have caused inflammation or pus in the antrum of Highmore. Out of this number, from my own records, only one had pus in the antrum, as far as examination could reveal; and this is the only marked and certain case of empyema of the maxillary sinus caused by the teeth that I have seen. I have, on the other hand, treated a case in which three teeth *were made pulpless by a diseased antrum*, and this I believe to be a condition more frequently brought about than the reverse, from the fact that some of the teeth in the skulls examined perforated the floor of the antrum with no protuberance, septa, or other covering save that of the mucous membrane. (See Figures 1 and 2.)

Such perforation I have found in eight cases, which number I believe to be smaller than it should be, could all cases have been thoroughly examined for minute openings. It must be evident that the mucous membrane covering the apices of such teeth, if diseased, could easily cause the death of a tooth by destruction of its blood and nerve supply, which, in its turn, would produce a dental abscess or inflammation about the roots of the tooth, thus producing a difficult case to diagnose. The possibilities are that such diseased teeth having been found connected with the antrum, the teeth have borne the blame, instead of the diagnosis having been properly made, and the opinion has

*NOTE.—It is so difficult to reproduce these sections by cuts, that the specimens themselves must be seen, in order to thoroughly comprehend many of the points referred to in the paper.

prevailed that diseased teeth are largely the *cause* of antral trouble instead of the *result*. In order that these cases can be properly diagnosed, they must needs be examined by one thoroughly familiar with alveolar abscesses, and the causes leading thereto, which I claim very few persons can do who are not experienced dental practitioners of acute and accurate observation.

Fig. 3.

It would seem that these teeth whose roots perforate the antrum, where there is neither septa nor protuberance over the roots of the teeth, are more likely to cause the trouble in question than those which show the tubercle above the roots. This protuberance in question (pictured by Zuckerkandl, and copied by Bosworth, photographs of which cut I show you), (See Fig. 4) seems to be the prevailing idea of the relation of the teeth to the floor of the antrum, but which from my own observation is quite erroneous, never having seen but one case which approaches this condition. (See Fig. 1-a.) You can readily see these tubercles are not in the center of the floor, nor in direct line, nor is the floor flat for their reception, as Zuckerkandl's picture shows it (See Fig. 4), consequently his illustration seems ideal rather than true to nature. It would seem the rational thing (if we are to treat diseases successfully) to first know the anatomy of the parts, then we have the true foundation upon which to build our idea of the pathology. These two points being founded on truth, our treatment is comparatively easy; whereas, if we proceed on an improper conception of the anatomy, we are endeavoring to treat imaginary things, and it is only through nature's kindness that our patients recover, for nature follows unchangeable laws.

Some further observations made in regard to the floor of the antrum are as follows: in about 25 per cent. of the cases examined, small septa or ridges were found to cross the antrum (See Fig. 2). These ridges, you will observe, have no relation to the position of the roots of the teeth, although in some cases they were found to come directly over the roots in place of the tubercle, as pictured by Zuckerkandl.

Fig. 4.

Another evidence can be presented to show that abscessed teeth do not frequently break into the antrum, by taking into consideration the amount of cancellous tissues found about and above the roots of the molar teeth in almost every case, and the very thin or entire absence of bone over the buccal surfaces of the roots. By examination of this section (See Fig. 3-a), it will be observed that the diploe or cancellous bone about the roots of most of the teeth affords quite ample space for the products of inflammation. These spaces are filled with soft tissues, like marrow, and this tissue easily takes on inflammation, and the products of inflammation may largely displace them. Again, when we have inflammation in this cancellous tissue, and about the apex of the root, we have the peridental membrane largely involved. This membrane gives way, as is evidenced by the lengthening of the tooth, and the products of inflammation may easily push down the side of a root, and also easily perforate the alveolar tissues, where they are very thin or where the bone is entirely absent. Whereas the floor of the antrum is usually thicker and of a dense, horny character, consequently it would not be perforated as quickly as the alveolar process. It seems in these cases that the peridental membrane is very largely affected, which is evidenced by the examination of the sockets and bone where there has been an alveolar abscess; this is readily seen in specimen 1, (See Fig 1) which you have already examined for another point. This specimen shows the evidence of a perforation in three separate places, one for each separate root, the two buccal roots having discharged the products of their inflammation in the mouth, and the palatal root opening into the antrum at the apex of the tubercle. This antrum also has a more distinct septum than most that were examined, although I found four or five septa that were even larger than this.

I found one case of which I especially wish to speak, and which has already been mentioned. Skull No. 81 of my examinations has evidence of the worst trouble I found from alveolar abscess. In this case the roots of the canine, the first and second bicuspid, and palatal roots of the first molar were involved. A cavity had formed in this case reaching from the lateral tooth back to the first molar, and from the alveolar border to the summit of the canine fossa, measuring about an inch in diameter.

A septum seemed to have formed or had already been formed between this cavity and the antrum, completely isolating the antrum from any connection with the trouble, although the palatal root of the first molar was involved; yet there seemed to have been no connection of the disease between this tooth and the antrum, although the floor of the antrum could easily have been perforated through the socket of the buccal roots of this same tooth. If there was a discharge from this molar at all it showed no evidence in the antrum, but did show evidence in this external and anterior cavity, which seemed to have been the result of chronic inflammation and collection of pus.

In regard to the proper place to perforate the antrum from the mouth when demanded: after taking the anatomy of the parts into consideration, it would seem that for several reasons the opening should be made between the apices of the second bicuspid and first molar.

1. Because this locality is the most accessible.

2. A perforation here does not interfere with the blood or nerve supply of either tooth.

3. By raising the lip well, and slanting the drill upward and backward, you are sure to strike the floor of the cavity almost at its lowest point.

4. If a tube must be inserted, in this position it will be held somewhat in place by the lip, whether the teeth are present or absent.

The summing up or rationale, then, of the evidence herewith seems to be:

1. That the anatomic relation between the teeth and the antrum is not generally understood, since the sections here shown give evidence of much more cancellous bone than is usually considered to exist.

2. Small septa are present in a large per cent. of cases, and these septa or ridges have no direct relation to the position of the teeth.

3. The evidence seems to indicate that the protrusion of the teeth into the cavity is very largely the exception, instead of the rule, and that if they do protrude it is not evidence that an alveolar abscess would break there, since these tubercles are usually formed of dense, hard bone.

4. A number of skulls have been found where there is a perforation of the bone by the apices of the teeth and no protrusion, but that these apices are simply covered with mucous membrane; thereby the teeth may be affected by inflammation of the antrum, causing their death and loss, or there may be a continuance of the trouble in the antrum by their presence, in consequence of this special feature of the anatomy.

5. That pulpless and inflamed teeth are thought to be the usual cause of antral trouble where the reverse is oftener probably the case.

6. Statistics seem to show that a very small per cent. of abscessed teeth have any connection whatever with the antrum—this per cent. probably not being over seven to ten.

7. That seemingly the best place to perforate the antrum for pus, is between the apices of the second bicuspid and first molar.

Since writing the above, I have examined an additional 400 skulls and find the figures changed in regard to the per cent. of abscessed molars which were connected with the antrum. In 500 skulls (*making one thousand antra*) I find 252 upper molars abscessed—making 25 per cent. of antra which have abscesses in this locality or every fourth antrum; this per cent. is probably smaller than it should be, since many teeth were lost and the alveolar process absorbed away, and undoubtedly some of these lost teeth had been abscessed. Out of these 252 possible cases, perforation into the antrum was found only 12 times, thus showing over $4\frac{1}{2}$ per cent. or about one in every 21 of the abscessed teeth in this locality which communicated with the antrum.

### DISCUSSION.

Dr. Talbot said, having in the last fifteen years made over twenty thousand examinations of skulls, he had found some of the conditions noted by the essayist. Of the skulls of civilized persons he had noticed many of these separations of the antrum, but he had never seen such in ancient skulls or the skulls of pure races. There is a state of things not brought before you by the paper; it is arrest of development of the bones of the face. This is a condition very common, in civilized cities especially. In these cases the antrum is about half the size of the antrum in pure races, and it is such cases where protrusions occur, as shown in the specimens exhibited. He said he would like to ask how the treatment would be carried out where a septum nearly closed the cavity? Would there not have to be two openings? There is much variation in the shape and size of this cavity in different skulls, and it would be well if they were classified as to shape and size and other peculiarities. He had found a number of cases where the opening from the antrum into the nose had been closed up, owing to the arrest of development of the bone.

Dr. Latham said every one knows the difficulty of deciding where to open into the antrum, but she thought the best place was from the socket of the first or second molar, when these teeth are missing or can be extracted. A good way to get rid of the pus, when the patient will help you, is by means of constant irrigation, that is by washing out with the syringe ten or a dozen times a day.

Dr. Clifford said he was impressed with the desirability in cases of antral trouble not to depend upon local treatment. In many cases by treating systemically and getting the system in first rate condition the catarrh will dry up without other treatment. In cases of purulent secretions of any nature, or affecting any part of the system, constitutional treatment is of the greatest importance.

Dr. Fletcher in closing the discussion, said that he had not aimed at going into the question of treatment in his paper, but had wished to present the case in a pathologic and anatomic light. We should find out what a diseased condition is before we undertake to treat it. In regard to the septum in the antrum, he said if the perforation was made just where indicated, and the patient assumed a horizontal position, every part of the antrum would drain, except in a case where the septum extended clear or nearly across, when, of course, there would have to be another perforation. If the first molar is absent the best place for the perforation would be through its socket. In every case he had examined, this would enter the antrum. In treatment he favored both constitutional and local treatment. Some cases are very tedious. He had one now which he had been treating for five months and it was not well yet.

## Figures and Tables

**Fig. 1. f1:**
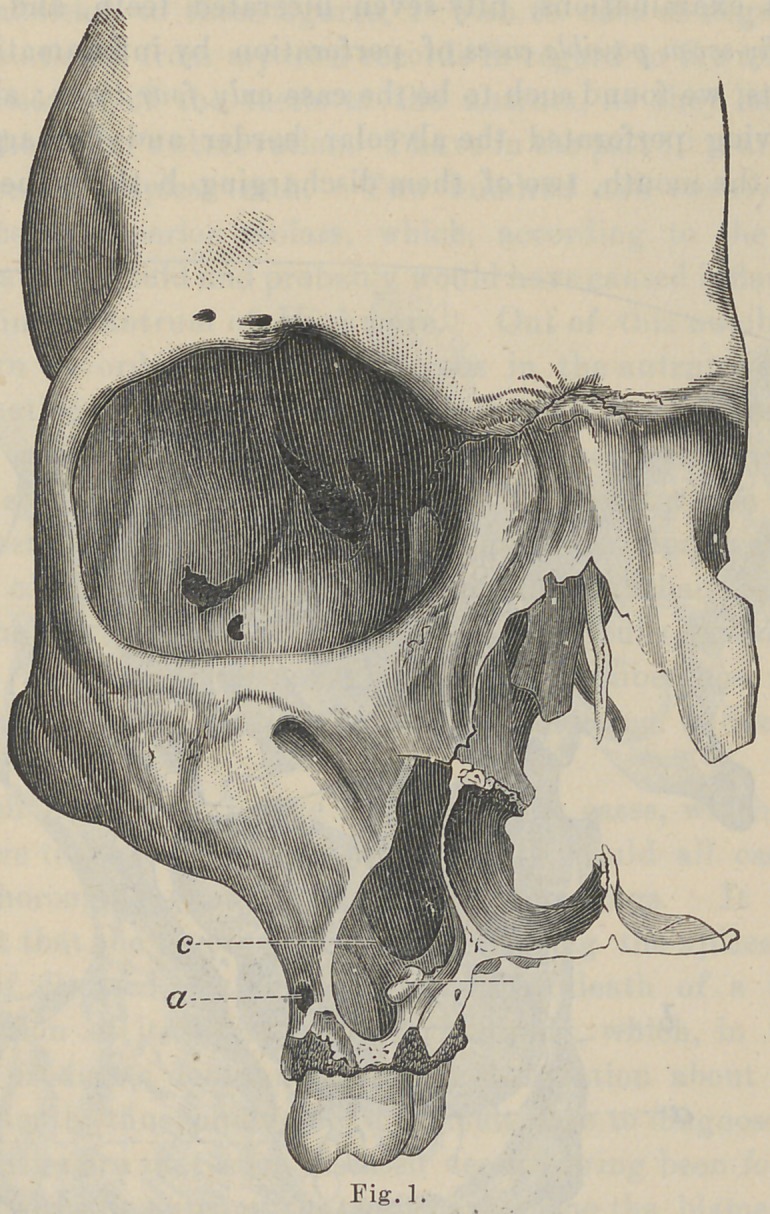


**Fig. 2. f2:**
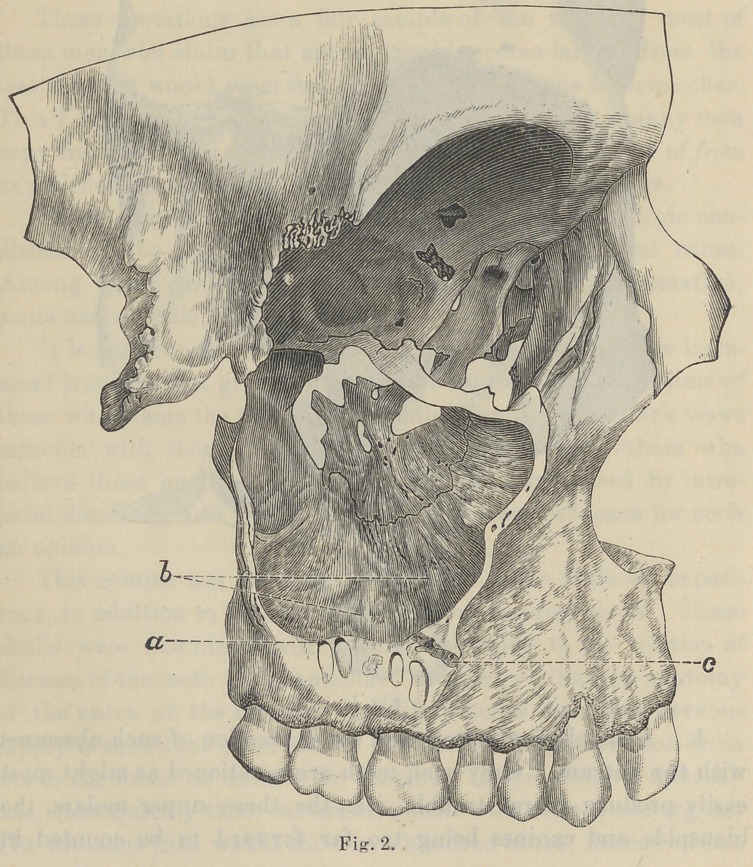


**Fig. 3. f3:**
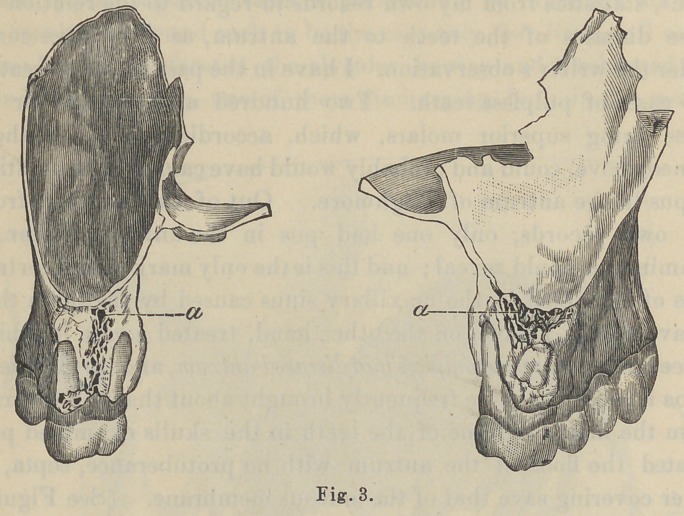


**Fig. 4. f4:**